# Supplementary material for: Polyphenol supplementation and executive functioning in overweight and obese adults at risk of cognitive impairment: A systematic review and meta-analysis
Source: PLoS One. 2023 May 25;18(5):e0286143. doi: 10.1371/journal.pone.0286143 (PMC10212191; doi:10.1371/journal.pone.0286143)
Supplement: S1 Table — (DOCX) [file pone.0286143.s002.docx]

**Protocol**

**Research Question:**

Does the acute/chronic intake of polyphenols rich diet/supplementation have a positive effect on executive functions in obese/overweight population?

**Inclusion criteria:**

- Obese/ overweight aadults of any age.
- Original article
- Peer reviewed articles in English

| Research question | Does the acute/chronic intake of polyphenols rich diet/supplementation have a positive effect on executive functions in obese/overweight population?? |
| --- | --- |
| Population | Adult age>18 |
| Intervention | Acute and/or chronic (poly)phenols-rich supplementation |
| Comparator | Any: food, juice, placebo… |
| Outcome | Executive functions |
| Study design and setting | RCT |

**Exclusion criteria:**

- Studies written in languages other than English
- Data from congress or workshop publications
- Animal studies
- Studies in which no supplementation was given
- Studies which administered multiple supplements in addition to (poly)phenol
- Studies conducted in populations diagnosed with severe cognitive impairment/dementia
- Methodological deficiencies (e.g., allocation not randomized, absence of control comparison (e.g., Placebo (PLA) or very low poly(phenols) dose and/or content,etc.), participant not blinded, and inappropriate statistical analysis procedures),
- Case studies, encyclopedias, book chapters, and reviews were excluded, although the bibliographies of the latter were consulted to refine article searches

**Data Sources and Search Strategy**

Data bases: Pubmed, Psychinfo, Scopus, Cochrane trials library

Search terms:

Polyphenols and cognition component will be combined with AND

| Polyphenols component | Cognition component |
| --- | --- |
| pomegranate or polyphenol or flavonoids, or polyphenolic compound, or polyphenolic compounds, or isoflavone, or flavanol, or phytoestrogen, or resveratrol, or ellagitannin, or ellagic acid, or punicalagin, or anthocyanins, or proanthocyanidin or proanthocyanidins. | mild cognitive impairment or MCI, or cognition, or cognitive performance, or cognitive function, or brain function, or executive function, or neuroimaging, or neural, or magnetic resonance imaging, or MRI, or fMRI, or grey matter, or gray matter, or brain structure, or electrophysiology, or EEG, or event related potential, or neuroblast, or cerebral blood flow, or CBF, or regional perfusion, or pulsatility index, or transcranial doppler, or TCD, or near-infrared spectroscopy, or NIRS, or total haemoglobin, or oxygenated haemoglobin, or oxy-Hb, or deoxygenated haemoglobin, or Deoxy-Hb. |
