# Supplementary material for: Polyphenol supplementation and executive functioning in overweight and obese adults at risk of cognitive impairment: A systematic review and meta-analysis
Source: PLoS One. 2023 May 25;18(5):e0286143. doi: 10.1371/journal.pone.0286143 (PMC10212191; doi:10.1371/journal.pone.0286143)
Supplement: S2 Table — (DOCX) [file pone.0286143.s003.docx]

Table S1

Outcomes of the sensitivity analyses for the Level Two meta-analyses

| RCTs | Model statistics with this study omitted | | |
| --- | --- | --- | --- |
| Study omitted | Hedges *g* | *z* | *P* |
| Fournier et al. 2007 | 0.10 | 1.59 | 0.11 |
| Sala-vila et al. 2020 | 0.12 | 1.66 | 0.095 |
| Ahles et al. 2020 | 0.10 | 1.56 | 0.11 |
| Kreijkamp-Kaspar et al. 2004 | 0.11 | 1.58 | 0.11 |
| Anton et al. 2018 | 0.10 | 1.51 | 0.12 |
| Henderson et al. 2012 | 0.10 | 1.60 | 0.10 |
| Huhn et al. 2018 | 0.10 | 1.61 | 0.096 |
| Bowtell et al. 2017 | 0.094 | 1.38 | 0.16 |
| Herrlinger et al., 2018 | 0.05 | 0.96 | 0.33 |
| Cox et al. 2014 | 0.05 | 0.94 | 0.34 |
| Boespflug et al. 2017 | 0.098 | 1.45 | 0.14 |
| Evans et al. 2017 | 0.03 | 0.73 | 0.46 |
| You et al. 2021 | 0.08 | 1.28 | 0.19 |
| Krikorian et al. 2022 | 0.08 | 1.24 | 0.21 |
| Yahya et al. 2017 | 0.09 | 1.34 | 0.17 |
| Crossover trials | Model statistics with this study omitted | | |
| Study omitted | Hedges *g* | *z* | *P* |
| Wong et al. 2013 | 0.005 | 0.046 | 0.96 |
| Kennedy et al. 2017 | 0.09 | 0.85 | 0.39 |
| Dodd et al. 2019 | 0.10 | 0.94 | 0.34 |
| Cook et al. 2020 | 0.08 | 0.82 | 0.41 |
| Alharbi et al. 2016 | 0.08 | 0.74 | 0.45 |
| Bondonno et al. 2020 | 0.10 | 0.93 | 0.34 |
| Igwea et al. 2020 | 0.11 | 1.07 | 0.28 |
| Keane et al. 2016 | 0.11 | 1.07 | 0.28 |
