# Supplementary material for: Polyphenol supplementation and executive functioning in overweight and obese adults at risk of cognitive impairment: A systematic review and meta-analysis
Source: PLoS One. 2023 May 25;18(5):e0286143. doi: 10.1371/journal.pone.0286143 (PMC10212191; doi:10.1371/journal.pone.0286143)
Supplement: S3 Table — (PDF) [file pone.0286143.s004.pdf]

	Study name	Comparison
1	Wong et al. 2013	Color naming trial (Seconds)
2	Wong et al. 2013	Word reading trial (Seconds)
3	Kennedy et al. 2017	Color naming trial (Seconds) at 1 hour 1600mg A
4	Kennedy et al. 2017	Color naming trial (Seconds) at 1 hour 1600mg B
5	Kennedy et al. 2017	Color naming trial (Seconds) at 1 hour 800mg A
6	Kennedy et al. 2017	Color naming trial (Seconds) at 1 hour 800mg B
7	Kennedy et al. 2017	Color naming trial (Seconds) at 2.5 hours 1600mg A
8	Kennedy et al. 2017	Color naming trial (Seconds) at 2.5 hours 1600mg B
9	Kennedy et al. 2017	Color naming trial (Seconds) at 2.5 hours 800m A
10	Kennedy et al. 2017	Color naming trial (Seconds) at 2.5 hours 800mg B
11	Kennedy et al. 2017	Color naming trial (Seconds) at 4 hours 1600mg A
12	Kennedy et al. 2017	Color naming trial (Seconds) at 4 hours 1600mg B
13	Kennedy et al. 2017	Color naming trial (Seconds) at 4 hours 800mg A
14	Kennedy et al. 2017	Color naming trial (Seconds) at 4 hours 800mg B
15	Kennedy et al. 2017	Color naming trial (Seconds) at 6 hours 1600mg B
16	Kennedy et al. 2017	Color naming trial (Seconds) at 6 hours 1600mg A
17	Kennedy et al. 2017	Color naming trial (Seconds) at 6 hours 800mg A
18	Kennedy et al. 2017	Color naming trial (Seconds) at 6 hours 800mg B
19	Kennedy et al. 2017	Corsi Blockaat 1 hour 1600mg A
20	Kennedy et al. 2017	Corsi Blockaat 1 hour 1600mg B
21	Kennedy et al. 2017	Corsi Blockaat 1 hour 800mg A
22	Kennedy et al. 2017	Corsi Blockaat 1 hour 800mg B
23	Kennedy et al. 2017	Corsi Blockaat 2.5 hour 1600mg A
24	Kennedy et al. 2017	Corsi Blockaat 2.5 hour 1600mg B
25	Kennedy et al. 2017	Corsi Blockaat 2.5 hour 800mg A
26	Kennedy et al. 2017	Corsi Blockaat 2.5 hour 800mg B
27	Kennedy et al. 2017	Corsi Blockaat 4 hour 1600mg A
28	Kennedy et al. 2017	Corsi Blockaat 4 hour 1600mg B
29	Kennedy et al. 2017	Corsi Blockaat 4 hour 800mg A
30	Kennedy et al. 2017	Corsi Blockaat 4 hour 800mg B
31	Kennedy et al. 2017	Corsi Blockaat 6 hour 1600mg A
32	Kennedy et al. 2017	Corsi Blockaat 6 hour 1600mg B
33	Kennedy et al. 2017	Corsi Blockaat 6 hour 800mg A
34	Kennedy et al. 2017	Corsi Blockaat 6 hour 800mg B
35	Kennedy et al. 2017	Peg and Ball task completion time msec 1600mg at 1 hour A
36	Kennedy et al. 2017	Peg and Ball task completion time msec 1600mg at 1 hour B
37	Kennedy et al. 2017	Peg and Ball task completion time msec 1600mg at 2,5 hour A
38	Kennedy et al. 2017	Peg and Ball task completion time msec 1600mg at 2,5 hour B
39	Kennedy et al. 2017	Peg and Ball task completion time msec 1600mg at 4 hour A

	Study design	age	Polyphenol dose mg	Dose	Polyphenol group	Duration in days	Duration	Data format	Difference in means	Standard Error
1	CO	Over 60	75.000	Below	Resveratrol	84.000	Chronic	Independent groups (means, SD's)		
2	CO	Over 60	75.000	Below	Resveratrol	84.000	Chronic	Independent groups (means, SD's)		
3	CO	Below 60	1600.000	Above	Flavonoid	0.041	Acute	Independent groups (means, SD's)		
4	CO	Below 60	1600.000	Above	Flavonoid	0.041	Acute	Independent groups (means, SD's)		
5	CO	Below 60	800.000	Above	Flavonoid	0.041	Acute	Independent groups (means, SD's)		
6	CO	Below 60	800.000	Above	Flavonoid	0.041	Acute	Independent groups (means, SD's)		
7	CO	Below 60	1600.000	Above	Flavonoid	0.100	Acute	Independent groups (means, SD's)		
8	CO	Below 60	1600.000	Above	Flavonoid	0.100	Acute	Independent groups (means, SD's)		
9	CO	Below 60	800.000	Above	Flavonoid	0.100	Acute	Independent groups (means, SD's)		
10	CO	Below 60	800.000	Above	Flavonoid	0.100	Acute	Independent groups (means, SD's)		
11	CO	Below 60	1600.000	Above	Flavonoid	0.166	Acute	Independent groups (means, SD's)		
12	CO	Below 60	1600.000	Above	Flavonoid	0.166	Acute	Independent groups (means, SD's)		
13	CO	Below 60	800.000	Above	Flavonoid	0.166	Acute	Independent groups (means, SD's)		
14	CO	Below 60	800.000	Above	Flavonoid	0.166	Acute	Independent groups (means, SD's)		
15	CO	Below 60	1600.000	Above	Flavonoid	0.250	Acute	Independent groups (means, SD's)		
16	CO	Below 60	1600.000	Above	Flavonoid	0.250	Acute	Independent groups (means, SD's)		
17	CO	Below 60	800.000	Above	Flavonoid	0.250	Acute	Independent groups (means, SD's)		
18	CO	Below 60	800.000	Above	Flavonoid	0.250	Acute	Independent groups (means, SD's)		
19	CO	Below 60	1600.000	Above	Flavonoid	0.041	Acute	Independent groups (means, SD's)		
20	CO	Below 60	1600.000	Above	Flavonoid	0.041	Acute	Independent groups (means, SD's)		
21	CO	Below 60	800.000	Above	Flavonoid	0.041	Acute	Independent groups (Sample size, t)		
22	CO	Below 60	800.000	Above	Flavonoid	0.041	Acute	Independent groups (means, SD's)		
23	CO	Below 60	1600.000	Above	Flavonoid	0.100	Acute	Independent groups (means, SD's)		
24	CO	Below 60	1600.000	Above	Flavonoid	0.100	Acute	Independent groups (means, SD's)		
25	CO	Below 60	800.000	Above	Flavonoid	0.100	Acute	Independent groups (means, SD's)		
26	CO	Below 60	800.000	Above	Flavonoid	0.100	Acute	Independent groups (means, SD's)		
27	CO	Below 60	1600.000	Above	Flavonoid	0.166	Acute	Independent groups (means, SD's)		
28	CO	Below 60	1600.000	Above	Flavonoid	0.166	Acute	Independent groups (means, SD's)		
29	CO	Below 60	800.000	Above	Flavonoid	0.166	Acute	Independent groups (means, SD's)		
30	CO	Below 60	800.000	Above	Flavonoid	0.166	Acute	Independent groups (means, SD's)		
31	CO	Below 60	1600.000	Above	Flavonoid	0.250	Acute	Independent groups (means, SD's)		
32	CO	Below 60	1600.000	Above	Flavonoid	0.250	Acute	Independent groups (means, SD's)		
33	CO	Below 60	800.000	Above	Flavonoid	0.250	Acute	Independent groups (means, SD's)		
34	CO	Below 60	800.000	Above	Flavonoid	0.250	Acute	Independent groups (means, SD's)		
35	CO	Below 60	1600.000	Above	Flavonoid	0.041	Acute	Independent groups (means, SD's)		
36	CO	Below 60	1600.000	Above	Flavonoid	0.041	Acute	Independent groups (means, SD's)		
37	CO	Below 60	1600.000	Above	Flavonoid	0.100	Acute	Independent groups (means, SD's)		
38	CO	Below 60	1600.000	Above	Flavonoid	0.100	Acute	Independent groups (means, SD's)		
39	CO	Below 60	1600.000	Above	Flavonoid	0.166	Acute	Independent groups (means, SD's)		

	Intervention N (Optional)	Control N (Optional)	Effect direction	Std diff in means	Std Err	Hedges's g	Std Err	Difference in means	Std Err	X
1				0.256	0.268	0.252	0.265	2.100	2.194	
2				0.962	0.282	0.948	0.278	5.000	1.390	
3				-0.149	0.221	-0.148	0.219	-12.100	17.878	
4				-0.183	0.221	-0.182	0.219	-9.700	11.687	
5				0.015	0.221	0.015	0.219	0.800	12.012	
6				0.156	0.221	0.155	0.219	8.000	11.297	
7				-0.152	0.221	-0.151	0.219	-9.500	13.800	
8				-0.435	0.223	-0.431	0.221	-22.200	11.271	
9				0.119	0.221	0.118	0.219	5.500	10.241	
10				-0.094	0.221	-0.093	0.219	-4.500	10.588	
11				-0.005	0.221	-0.005	0.219	-0.300	12.099	
12				-0.152	0.221	-0.151	0.219	-12.000	17.425	
13				0.379	0.223	0.376	0.221	19.200	11.178	
14				-0.012	0.221	-0.012	0.219	-0.600	11.126	
15				-0.056	0.278	-0.055	0.273	-30.000	147.901	
16				0.300	0.222	0.297	0.220	16.300	11.996	
17				0.333	0.222	0.330	0.220	18.500	12.259	
18				0.219	0.222	0.217	0.219	14.900	15.025	
19				0.364	0.220	0.361	0.218	3.150	1.889	
20				0.074	0.218	0.074	0.216	0.630	1.846	
21										
22				0.029	0.218	0.029	0.216	0.240	1.818	
23				0.009	0.218	0.008	0.216	0.070	1.793	
24				-0.282	0.219	-0.280	0.217	-2.400	1.854	
25				-0.134	0.218	-0.133	0.216	-1.150	1.868	
26				-0.108	0.218	-0.107	0.216	-0.910	1.841	
27				-0.103	0.218	-0.102	0.216	-0.830	1.761	
28				-0.440	0.221	-0.436	0.219	-3.380	1.676	
29				0.243	0.219	0.241	0.217	2.130	1.915	
30				0.070	0.218	0.069	0.216	0.560	1.746	
31				0.431	0.221	0.427	0.219	3.420	1.732	
32				0.196	0.219	0.194	0.217	1.760	1.960	
33				-0.021	0.218	-0.021	0.216	-0.170	1.790	
34				-0.040	0.218	-0.040	0.216	-0.370	2.001	
35				0.114	0.221	0.113	0.219	172.000	332.904	
36				-0.095	0.221	-0.094	0.219	-142.000	329.574	
37				0.103	0.221	0.102	0.219	150.000	320.295	
38				0.082	0.221	0.081	0.219	110.000	296.853	
39				0.015	0.221	0.015	0.219	21.000	312.106	

	Study name	Comparison
40	Kennedy et al. 2017	Peg and Ball task completion time msec 1600mg at 4 hour B
41	Kennedy et al. 2017	Peg and Ball task completion time msec 1600mg at 6 hour A
42	Kennedy et al. 2017	Peg and Ball task completion time msec 1600mg at 6 hour B
43	Kennedy et al. 2017	Peg and Ball task completion time msec 800mg p value
44	Kennedy et al. 2017	Peg and Ball task number of error 1600mg at 1 hour A
45	Kennedy et al. 2017	Peg and Ball task number of error 1600mg at 1 hour B
46	Kennedy et al. 2017	Peg and Ball task number of error 1600mg at 2.5 hour A
47	Kennedy et al. 2017	Peg and Ball task number of error 1600mg at 2.5 hour B
48	Kennedy et al. 2017	Peg and Ball task number of error 1600mg at 4 hour A
49	Kennedy et al. 2017	Peg and Ball task number of error 1600mg at 4 hour B
50	Kennedy et al. 2017	Peg and Ball task number of error 1600mg at 6 hour A
51	Kennedy et al. 2017	Peg and Ball task number of error 1600mg at 6 hour B
52	Kennedy et al. 2017	Peg and Ball task number of error 800mg at 1 hour A
53	Kennedy et al. 2017	Peg and Ball task number of error 800mg at 1 hour B
54	Kennedy et al. 2017	Peg and Ball task number of error 800mg at 2.5 hour A
55	Kennedy et al. 2017	Peg and Ball task number of error 800mg at 2.5 hour B
56	Kennedy et al. 2017	Peg and Ball task number of error 800mg at 4 hour A
57	Kennedy et al. 2017	Peg and Ball task number of error 800mg at 4 hour B
58	Kennedy et al. 2017	Peg and Ball task number of error 800mg at 6 hour A
59	Kennedy et al. 2017	Peg and Ball task number of error 800mg at 6 hour B
60	Kennedy et al. 2017	Peg and Ball task thinking time msec 1600mg p value
61	Kennedy et al. 2017	Peg and Ball task thinking time msec 800mg p value
62	Fournier et al. 2007	Color matching task reaction time Soy milk
63	Fournier et al. 2007	Color matching task reaction time Soy supplement
64	Fournier et al. 2007	Color matching task total Error Soy milk
65	Fournier et al. 2007	Color matching task total Error Soy supplement
66	Fournier et al. 2007	Color naming trial (Seconds) soy milk (compatiblel) A
67	Fournier et al. 2007	Color naming trial (Seconds) soy milk (Incompatible) B
68	Fournier et al. 2007	Color naming trial (Seconds) soy milk (neutral) C
69	Fournier et al. 2007	Color naming trial (Seconds) soy supplement (compatiblel) A
70	Fournier et al. 2007	Color naming trial (Seconds) soy supplement (Incompatible) B
71	Fournier et al. 2007	Color naming trial (Seconds) soy supplement (neutral) C
72	Fournier et al. 2007	Corsi Block Soy Milk
73	Fournier et al. 2007	Corsi Block Soy supplement
74	Dodd et al. 2019	Color naming trial (Seconds) A
75	Dodd et al. 2019	Color naming trial (Seconds) B
76	Dodd et al. 2019	Continuous performance task A
77	Dodd et al. 2019	Continuous performance task B
78	Dodd et al. 2019	Digit Switch (switch cost) A

	Study design	age	Polyphenol dose mg	Dose	Polyphenol group	Duration in days	Duration	Data format	Difference in means	Standard Error
40	CO	Below 60	1600.000	Above	Flavonoid	0.166	Acute	Independent groups (means, SD's)		
41	CO	Below 60	1600.000	Above	Flavonoid	0.250	Acute	Independent groups (means, SD's)		
42	CO	Below 60	1600.000	Above	Flavonoid	0.250	Acute	Independent groups (means, SD's)		
43	CO	Below 60	800.000	Above	Flavonoid	0.250	Acute	Independent groups (Sample size, p)		
44	CO	Below 60	1600.000	Above	Flavonoid	0.041	Acute	Independent groups (means, SD's)		
45	CO	Below 60	1600.000	Above	Flavonoid	0.041	Acute	Independent groups (means, SD's)		
46	CO	Below 60	1600.000	Above	Flavonoid	0.100	Acute	Independent groups (means, SD's)		
47	CO	Below 60	1600.000	Above	Flavonoid	0.100	Acute	Independent groups (means, SD's)		
48	CO	Below 60	1600.000	Above	Flavonoid	0.166	Acute	Independent groups (means, SD's)		
49	CO	Below 60	1600.000	Above	Flavonoid	0.166	Acute	Independent groups (means, SD's)		
50	CO	Below 60	1600.000	Above	Flavonoid	0.250	Acute	Independent groups (means, SD's)		
51	CO	Below 60	1600.000	Above	Flavonoid	0.250	Acute	Independent groups (means, SD's)		
52	CO	Below 60	800.000	Above	Flavonoid	0.041	Acute	Independent groups (means, SD's)		
53	CO	Below 60	800.000	Above	Flavonoid	0.041	Acute	Independent groups (means, SD's)		
54	CO	Below 60	800.000	Above	Flavonoid	0.100	Acute	Independent groups (means, SD's)		
55	CO	Below 60	800.000	Above	Flavonoid	0.100	Acute	Independent groups (means, SD's)		
56	CO	Below 60	800.000	Above	Flavonoid	0.166	Acute	Independent groups (means, SD's)		
57	CO	Below 60	800.000	Above	Flavonoid	0.166	Acute	Independent groups (means, SD's)		
58	CO	Below 60	800.000	Above	Flavonoid	0.250	Acute	Independent groups (means, SD's)		
59	CO	Below 60	800.000	Above	Flavonoid	0.250	Acute	Independent groups (means, SD's)		
60	CO	Below 60	1600.000	Above	Flavonoid	0.250	Acute	Independent groups (Sample size, p)		
61	BTW-P	Below 60	800.000	Above	Flavonoid	0.250	Acute	Independent groups (Sample size, p)		
62	BTW-P	Below 60	70.000	Below	Isoflavone	112.000	Chronic	Independent groups (means, SD's)		
63	BTW-P	Below 60	71.000	Below	Isoflavone	112.000	Chronic	Independent groups (means, SD's)		
64	BTW-P	Below 60	70.000	Below	Isoflavone	112.000	Chronic	Independent groups (means, SD's)		
65	BTW-P	Below 60	71.000	Below	Isoflavone	112.000	Chronic	Independent groups (means, SD's)		
66	BTW-P	Below 60	70.000	Below	Isoflavone	112.000	Chronic	Independent groups (means, SD's)		
67	BTW-P	Below 60	70.000	Below	Isoflavone	112.000	Chronic	Independent groups (means, SD's)		
68	BTW-P	Below 60	70.000	Below	Isoflavone	112.000	Chronic	Independent groups (means, SD's)		
69	BTW-P	Below 60	71.000	Below	Isoflavone	112.000	Chronic	Independent groups (means, SD's)		
70	BTW-P	Below 60	71.000	Below	Isoflavone	112.000	Chronic	Independent groups (means, SD's)		
71	BTW-P	Below 60	71.000	Below	Isoflavone	112.000	Chronic	Independent groups (means, SD's)		
72	BTW-P	Below 60	70.000	Below	Isoflavone	112.000	Chronic	Independent groups (means, SD's)		
73	BTW-P	Below 60	71.000	Below	Isoflavone	112.000	Chronic	Independent groups (means, SD's)		
74	CO	Over 60	579.000	Above	Flavonoid	0.041	Acute	Independent groups (means, SD's)		
75	CO	Over 60	579.000	Above	Flavonoid	0.200	Acute	Independent groups (means, SD's)		
76	CO	Over 60	579.000	Above	Flavonoid	0.041	Acute	Independent groups (means, SD's)		
77	CO	Over 60	579.000	Above	Flavonoid	0.200	Acute	Independent groups (means, SD's)		
78	CO	Over 60	579.000	Above	Flavonoid	0.041	Acute	Independent groups (means, SD's)		

	Intervention N (Optional)	Control N (Optional)	Effect direction	Std diff in means	Std Err	Hedges's g	Std Err	Difference in means	Std Err	X
40				0.178	0.221	0.176	0.219	244.000	303.268	
41				-0.079	0.221	-0.078	0.219	-114.000	318.813	
42				0.011	0.221	0.011	0.219	15.000	300.375	
43				0.591	0.226	0.586	0.224			
44				-0.403	0.223	-0.399	0.221	-1.120	0.614	
45				0.240	0.222	0.237	0.220	0.690	0.636	
46				-0.033	0.221	-0.032	0.219	-0.100	0.675	
47				0.050	0.221	0.050	0.219	0.150	0.659	
48				-0.022	0.221	-0.021	0.219	-0.060	0.614	
49				-0.143	0.221	-0.142	0.219	-0.470	0.727	
50				0.141	0.221	0.139	0.219	0.400	0.628	
51				-0.208	0.221	-0.206	0.219	-0.740	0.787	
52				-0.007	0.221	-0.007	0.219	-0.020	0.656	
53				-0.008	0.221	-0.008	0.219	-0.020	0.539	
54				0.097	0.221	0.096	0.219	0.370	0.840	
55				0.082	0.221	0.082	0.219	0.300	0.804	
56				0.132	0.221	0.131	0.219	0.410	0.685	
57				-0.105	0.221	-0.104	0.219	-0.300	0.629	
58				0.120	0.221	0.119	0.219	0.450	0.826	
59				0.484	0.224	0.479	0.222	1.600	0.731	
60				0.601	0.226	0.595	0.224			
61				0.638	0.226	0.632	0.224			
62				-0.164	0.334	-0.160	0.327	-18.000	36.714	
63				-0.017	0.310	-0.016	0.304	-2.000	36.955	
64				-0.038	0.334	-0.037	0.326	-1.200	10.620	
65				-0.206	0.311	-0.202	0.305	-7.100	10.681	
66				-0.126	0.278	-0.124	0.274	-14.000	30.887	
67				0.197	0.278	0.194	0.274	17.000	23.898	
68				0.033	0.278	0.032	0.273	3.000	25.248	
69				-1.081	0.357	-1.057	0.349	-11.700	3.607	
70				0.467	0.281	0.460	0.277	36.000	21.377	
71				-1.404	0.372	-1.373	0.364	-14.550	3.453	
72				0.000	0.278	0.000	0.273	0.000	0.424	
73				0.065	0.272	0.064	0.268	0.100	0.422	
74				-0.335	0.279	-0.330	0.275	-14.550	12.055	
75				-0.245	0.222	-0.243	0.220	-11.260	10.139	
76				0.088	0.333	0.086	0.326	0.490	1.867	
77				-0.061	0.333	-0.060	0.326	-0.450	2.447	
78				0.633	0.342	0.619	0.334	83.720	44.058	

	Study name	Comparison
79	Dodd et al. 2019	Digit Switch (switch cost) B
80	Dodd et al. 2019	Digit Symbol Substitution Test A
81	Dodd et al. 2019	Digit Symbol Substitution Test B
82	Dodd et al. 2019	Go No Go (Seconds) A
83	Dodd et al. 2019	Go No Go (Seconds) B
84	Dodd et al. 2019	Letter Memory Total Correct A
85	Dodd et al. 2019	Letter Memory Total Correct B
86	Dodd et al. 2019	Location task A
87	Dodd et al. 2019	Location task B
88	Dodd et al. 2019	N-Back Percent correct A
89	Dodd et al. 2019	N-Back Percent correct B
90	Dodd et al. 2019	Random Word Generation A
91	Dodd et al. 2019	Random Word Generation B
92	Dodd et al. 2019	Three-Word Sets A
93	Dodd et al. 2019	Three-Word Sets B
94	Sala-vila et al. 2020	Block design
95	Sala-vila et al. 2020	Boston naming test
96	Sala-vila et al. 2020	Color naming trial (Seconds)
97	Sala-vila et al. 2020	TMT B (s)
98	Ahles et al. 2020	color and word trial (seconds)
99	Ahles et al. 2020	Number cross-out test accuracy
100	Ahles et al. 2020	Number cross-out test diligence
101	Kreijkamp-Kaspar et al. 2004	Bosoton naming task
102	Kreijkamp-Kaspar et al. 2004	Categorical verbal fluency A
103	Kreijkamp-Kaspar et al. 2004	Categorical verbal fluency Animal
104	Kreijkamp-Kaspar et al. 2004	Categorical verbal fluency N
105	Kreijkamp-Kaspar et al. 2004	Categorical verbal fluency Occupation
106	Kreijkamp-Kaspar et al. 2004	Digit Symbol Substitution Test
107	Kreijkamp-Kaspar et al. 2004	TMT B (s)
108	Anton et al. 2018	Controlled oral word association (Words generated) High-dose
109	Anton et al. 2018	Controlled oral word association (Words generated) Low-dose
110	Anton et al. 2018	Digit Symbol Substitution Test A
111	Anton et al. 2018	Digit Symbol Substitution Test B
112	Anton et al. 2018	Eriksen flanker task RT A
113	Anton et al. 2018	Eriksen flanker task RT B
114	Anton et al. 2018	Task switching accuracy A
115	Anton et al. 2018	Task switching accuracy B
116	Anton et al. 2018	Task switching switch RT (ms) B
117	Anton et al. 2018	Task switching switch RT (ms) A

	Study design	age	Polyphenol dose mg	Dose	Polyphenol group	Duration in days	Duration	Data format	Difference in means	Standard Error
79	CO	Over 60	579.000	Above	Flavonoid	0.200	Acute	Independent groups (means, SD's)		
80	CO	Over 60	579.000	Above	Flavonoid	0.041	Acute	Independent groups (means, SD's)		
81	CO	Over 60	579.000	Above	Flavonoid	0.200	Acute	Independent groups (means, SD's)		
82	CO	Over 60	579.000	Above	Flavonoid	0.041	Acute	Independent groups (means, SD's)		
83	CO	Over 60	579.000	Above	Flavonoid	0.200	Acute	Independent groups (means, SD's)		
84	CO	Over 60	579.000	Above	Flavonoid	0.041	Acute	Independent groups (means, SD's)		
85	CO	Over 60	579.000	Above	Flavonoid	0.200	Acute	Independent groups (means, SD's)		
86	CO	Over 60	579.000	Above	Flavonoid	0.041	Acute	Independent groups (means, SD's)		
87	CO	Over 60	579.000	Above	Flavonoid	0.200	Acute	Independent groups (means, SD's)		
88	CO	Over 60	579.000	Above	Flavonoid	0.041	Acute	Independent groups (means, SD's)		
89	CO	Over 60	579.000	Above	Flavonoid	0.200	Acute	Independent groups (means, SD's)		
90	CO	Over 60	579.000	Above	Flavonoid	0.041	Acute	Independent groups (means, SD's)		
91	CO	Over 60	579.000	Above	Flavonoid	0.200	Acute	Independent groups (means, SD's)		
92	CO	Over 60	579.000	Above	Flavonoid	0.041	Acute	Independent groups (means, SD's)		
93	CO	Over 60	579.000	Above	Flavonoid	0.200	Acute	Independent groups (means, SD's)		
94	BTW-P	Over 60	Not	Not	Unclassifia	730.500	Chronic	Independent groups (Sample size, p)		
95	BTW-P	Over 60	Not	Not	Unclassifia	730.500	Chronic	Independent groups (Sample size, p)		
96	BTW-P	Over 60	Not	Not	Unclassifia	730.500	Chronic	Independent groups (Sample size, p)		
97	BTW-P	Over 60	Not	Not	Unclassifia	730.480	Chronic	Independent groups (Sample size, p)		
98	BTW-P	Below 60	27.000	Below	Flavonoid	168.000	Chronic	Independent groups (Sample size, p)		
99	BTW-P	Below 60	27.000	Below	Flavonoid	168.000	Chronic	Independent groups (Sample size, p)		
100	BTW-P	Below 60	27.000	Below	Flavonoid	168.000	Chronic	Independent groups (Sample size, p)		
101	BTW-P	Over 60	99.000	Below	Isoflavone	365.240	Chronic	Independent groups (means, SD's)		
102	BTW-P	Over 60	99.000	Below	Isoflavone	365.240	Chronic	Independent groups (means, SD's)		
103	BTW-P	Over 60	99.000	Below	Isoflavone	365.240	Chronic	Independent groups (means, SD's)		
104	BTW-P	Over 60	99.000	Below	Isoflavone	365.240	Chronic	Independent groups (means, SD's)		
105	BTW-P	Over 60	99.000	Below	Isoflavone	365.240	Chronic	Independent groups (means, SD's)		
106	BTW-P	Over 60	99.000	Below	Isoflavone	365.240	Chronic	Independent groups (means, SD's)		
107	BTW-P	Over 60	99.000	Below	Isoflavone	365.240	Chronic	Independent groups (means, SD's)		
108	BTW-P	Over 60	1000.000	Above	Resveratrol	90.000	Chronic	Independent groups (means, SD's)		
109	BTW-P	Over 60	300.000	Below	Resveratrol	90.000	Chronic	Independent groups (means, SD's)		
110	BTW-P	Over 60	1000.000	Above	Resveratrol	90.000	Chronic	Cohen's d, SE		
111	BTW-P	Over 60	300.000	Below	Resveratrol	90.000	Chronic	Cohen's d, SE		
112	BTW-P	Over 60	1000.000	Above	Resveratrol	90.000	Chronic	Cohen's d, SE		
113	BTW-P	Over 60	300.000	Below	Resveratrol	90.000	Chronic	Cohen's d, SE		
114	BTW-P	Over 60	1000.000	Above	Resveratrol	90.000	Chronic	Cohen's d, SE		
115	BTW-P	Over 60	300.000	Below	Resveratrol	90.000	Chronic	Cohen's d, SE		
116	BTW-P	Over 60	300.000	Below	Resveratrol	90.000	Chronic	Cohen's d, SE		
117	BTW-P	Over 60	1000.000	Above	Resveratrol	90.000	Chronic	Cohen's d, SE		

	Intervention N (Optional)	Control N (Optional)	Effect direction	Std diff in means	Std Err	Hedges's g	Std Err	Difference in means	Std Err	X
79				0.279	0.335	0.273	0.328	47.240	56.447	
80				0.112	0.334	0.110	0.326	0.410	1.216	
81				0.130	0.334	0.127	0.326	0.580	1.485	
82				0.004	0.333	0.004	0.326	0.170	13.845	
83										
84				-0.033	0.333	-0.032	0.326	-0.530	5.371	
85				-0.123	0.334	-0.120	0.326	-0.750	2.040	
86				-0.524	0.339	-0.512	0.331	-1.890	1.202	
87				0.029	0.333	0.029	0.326	0.110	1.245	
88				-0.034	0.333	-0.033	0.326	-0.290	2.825	
89				0.007	0.333	0.007	0.326	0.250	12.492	
90				-0.043	0.333	-0.042	0.326	-0.450	3.484	
91				-0.063	0.333	-0.062	0.326	-0.870	4.601	
92				-0.004	0.333	-0.004	0.326	-0.020	1.749	
93				-0.302	0.335	-0.296	0.328	-0.570	0.628	
94				-0.095	0.078	-0.095	0.078			
95				-0.032	0.078	-0.032	0.078			
96				0.130	0.078	0.130	0.078			
97				-0.043	0.078	-0.043	0.078			
98				0.339	0.246	0.335	0.243			
99				-0.264	0.247	-0.261	0.244			
100				-0.322	0.248	-0.318	0.245			
101				-0.091	0.151	-0.091	0.151	-1.100	1.819	
102				0.173	0.151	0.172	0.151	0.400	0.350	
103				-0.272	0.152	-0.270	0.151	-1.100	0.612	
104				0.032	0.151	0.032	0.151	0.100	0.469	
105				-0.130	0.151	-0.129	0.151	-0.500	0.582	
106				0.085	0.151	0.085	0.151	0.900	1.597	
107				0.071	0.151	0.071	0.151	2.200	4.697	
108				-0.095	0.428	-0.091	0.412	-0.600	2.715	
109				-0.318	0.431	-0.305	0.414	-2.080	2.805	
110				0.700	0.300	0.670	0.287			
111				0.200	0.100	0.192	0.096			
112				-0.070	0.040	-0.067	0.038			
113				0.300	0.100	0.289	0.096			
114				0.500	0.240	0.479	0.230			
115				-0.420	0.210	-0.404	0.202			
116				-0.490	0.240	-0.471	0.231			
117				-0.910	0.410	-0.872	0.393			

	Study name	Comparison
118	Anton et al. 2018	TMT B (s) A
119	Anton et al. 2018	TMT B (s) B
120	Henderson et al. 2012	Block Design
121	Henderson et al. 2012	Boston Naming Test
122	Henderson et al. 2012	Category Fluency
123	Henderson et al. 2012	TMT B (s)
124	Huhn et al. 2018	TMT B (s)
125	Bowtell et al. 2017	Groton maze learning accuracy (total errors)
126	Bowtell et al. 2017	Groton maze learning moves/second
127	Bowtell et al. 2017	Identification speed
128	Bowtell et al. 2017	N 1 back reaction time
129	Bowtell et al. 2017	N 2 reaction time
130	Herrlinger et al., 2018	Spatial working memory
131	Herrlinger et al., 2018	Working memory
132	Cook et al. 2020	Paired associated learning variables
133	Cook et al. 2020	rapid visual information processing Latency (ms)
134	Cook et al. 2020	rapid visual information processing Probability of hit
135	Cook et al. 2020	rapid visual information processing Total false alarms
136	Cook et al. 2020	Spatial working memory errors
137	Cook et al. 2020	Spatial working memory strategy
138	Alharbi et al. 2016	Continuous Performance Task (errors) A
139	Alharbi et al. 2016	Continuous Performance Task (errors) B
140	Alharbi et al. 2016	Digit Symbol Substitution Test no. correct A
141	Alharbi et al. 2016	Digit Symbol Substitution Test no. correct B
142	Alharbi et al. 2016	Serial Sevens (number correct) A
143	Alharbi et al. 2016	Serial Sevens (number correct) B
144	Cox et al. 2014	Digit vigilance task A
145	Cox et al. 2014	Serial three subtraction task number of correct responses A
146	Cox et al. 2014	Serial three subtraction task number of correct responses B
147	Boespflug et al. 2017	N back Acc, 0-back %
148	Boespflug et al. 2017	N back Acc, 1-back %
149	Boespflug et al. 2017	N back Acc, 2-back %
150	Boespflug et al. 2017	N back RT, 0-back ms
151	Boespflug et al. 2017	N back RT, 1-back ms
152	Boespflug et al. 2017	N back RT, 2-back ms
153	Bondonno et al. 2020	RVIP correct
154	Bondonno et al. 2020	RVIP response time (ms)
155	Bondonno et al. 2020	Serial 3 subtractions correct
156	Bondonno et al. 2020	Serial 3 subtractions total number of substraction completed

	Study design	age	Polyphenol dose mg	Dose	Polyphenol group	Duration in days	Duration	Data format	Difference in means	Standard Error
118	BTW-P	Over 60	1000.000	Above	Resveratrol	90.000	Chronic	Cohen's d, SE		
119	BTW-P	Over 60	300.000	Below	Resveratrol	90.000	Chronic	Cohen's d, SE		
120	BTW-P	Over 60	91.000	Below	Isoflavone	913.100	Chronic	Independent groups (Sample size, p)		
121	BTW-P	Over 60	91.000	Below	Isoflavone	913.100	Chronic	Independent groups (Sample size, p)		
122	BTW-P	Over 60	91.000	Below	Isoflavone	913.100	Chronic	Independent groups (Sample size, p)		
123	BTW-P	Over 60	91.000	Below	Isoflavone	913.100	Chronic	Independent groups (Sample size, p)		
124	BTW-P	Over 60	200.000	Below	Resveratrol	180.000	Chronic	Independent groups (Sample size, p)		
125	BTW-P	Over 60	387.000	Below	Flavonoid	84.000	Chronic	Independent groups (means, SD's)		
126	BTW-P	Over 60	387.000	Below	Flavonoid	84.000	Chronic	Independent groups (means, SD's)		
127	BTW-P	Over 60	387.000	Below	Flavonoid	84.000	Chronic	Independent groups (means, SD's)		
128	BTW-P	Over 60	387.000	Below	Flavonoid	84.000	Chronic	Independent groups (Sample size, p)		
129	BTW-P	Over 60	387.000	Below	Flavonoid	84.000	Chronic	Independent groups (Sample size, p)		
130	BTW-P	Below 60	900.000	Above	Phenolic	90.000	Chronic	Independent groups (Sample size, t)		
131	BTW-P	Below 60	900.000	Above	Phenolic	90.000	Chronic	Independent groups (Sample size, t)		
132	CO	Over 60	210.000	Below	Flavonoid	168.000	Acute	Independent groups (means, SD's)		
133	CO	Over 60	210.000	Below	Flavonoid	168.000	Acute	Independent groups (means, SD's)		
134	CO	Over 60	210.000	Below	Flavonoid	168.000	Acute	Independent groups (means, SD's)		
135	CO	Over 60	210.000	Below	Flavonoid	168.000	Acute	Independent groups (means, SD's)		
136	CO	Over 60	210.000	Below	Flavonoid	168.000	Acute	Independent groups (means, SD's)		
137	CO	Over 60	210.000	Below	Flavonoid	168.000	Acute	Independent groups (means, SD's)		
138	CO	Below 60	272.000	Below	Flavonoid	0.083	Acute	Independent groups (means, SD's)		
139	CO	Below 60	272.000	Below	Flavonoid	0.250	Acute	Independent groups (means, SD's)		
140	CO	Below 60	272.000	Below	Flavonoid	0.083	Acute	Independent groups (means, SD's)		
141	CO	Below 60	272.000	Below	Flavonoid	0.250	Acute	Independent groups (means, SD's)		
142	CO	Below 60	272.000	Below	Flavonoid	0.083	Acute	Independent groups (means, SD's)		
143	CO	Below 60	272.000	Resveratrol	Flavonoid	0.250	Acute	Independent groups (means, SD's)		
144	BTW-P	Over 60	Not	Not	Unclassifia	0.041	Acute	Independent groups (Sample size, p)		
145	BTW-P	Over 60	Not	Not	Unclassifia	0.041	Acute	Independent groups (Sample size, p)		
146	BTW-P	Over 60	Not	Not	Unclassifia	28.000	Chronic	Independent groups (Sample size, p)		
147	BTW-P	Over 60	269.000	Below	Flavonoid	112.000	Chronic	Independent groups (means, SD's)		
148	BTW-P	Over 60	269.000	Below	Flavonoid	112.000	Chronic	Independent groups (means, SD's)		
149	BTW-P	Over 60	269.000	Below	Flavonoid	112.000	Chronic	Independent groups (means, SD's)		
150	BTW-P	Over 60	269.000	Below	Flavonoid	112.000	Chronic	Independent groups (means, SD's)		
151	BTW-P	Over 60	269.000	Below	Flavonoid	112.000	Chronic	Independent groups (means, SD's)		
152	BTW-P	Over 60	269.000	Below	Flavonoid	112.000	Chronic	Independent groups (means, SD's)		
153	CO	Over 60	4.890	Below	Flavonoid	0.166	Acute	Independent groups (means, SD's)		
154	CO	Over 60	4.890	Below	Flavonoid	0.166	Acute	Independent groups (means, SD's)		
155	CO	Over 60	4.890	Below	Flavonoid	0.166	Acute	Independent groups (means, SD's)		
156	CO	Over 60	4.890	Below	Flavonoid	0.166	Acute	Independent groups (means, SD's)		

	Intervention N (Optional)	Control N (Optional)	Effect direction	Std diff in means	Std Err	Hedges's g	Std Err	Difference in means	Std Err	X
118				0.200	0.080	0.192	0.077			
119				0.100	0.060	0.096	0.058			
120				0.031	0.115	0.031	0.115			
121				-0.050	0.115	-0.050	0.114			
122				-0.094	0.113	-0.093	0.113			
123				0.094	0.115	0.094	0.114			
124				-0.265	0.271	-0.261	0.267			
125				-0.253	0.395	-0.245	0.382	-4.900	7.629	
126				0.001	0.393	0.001	0.381	0.003	0.844	
127				-0.134	0.394	-0.130	0.381	-0.010	0.029	
128				0.686	0.405	0.664	0.392			
129				0.695	0.405	0.673	0.392			
130				0.524	0.263	0.517	0.259			
131				0.522	0.263	0.515	0.259			
132				0.543	0.385	0.527	0.374	3.000	2.087	
133				0.204	0.379	0.198	0.368	21.000	38.947	
134				0.202	0.379	0.196	0.368	0.040	0.075	
135				-0.035	0.378	-0.034	0.367	-0.180	1.951	
136				-0.110	0.378	-0.107	0.367	-1.000	3.423	
137				-0.125	0.378	-0.121	0.367	-1.000	3.024	
138				-0.245	0.290	-0.241	0.285	-1.500	1.764	
139				0.604	0.295	0.594	0.290	1.200	0.573	
140				0.132	0.289	0.130	0.284	1.200	2.623	
141				0.093	0.289	0.092	0.284	1.000	3.089	
142				0.090	0.289	0.089	0.284	1.900	6.079	
143				0.155	0.289	0.152	0.284	3.000	5.586	
144				0.519	0.263	0.512	0.259			
145				0.564	0.263	0.557	0.260			
146				0.532	0.263	0.525	0.259			
147				1.342	0.553	1.268	0.523	0.030	0.011	
148				0.199	0.501	0.188	0.474	0.020	0.050	
149				0.000	0.500	0.000	0.473	0.000	0.043	
150				-0.316	0.503	-0.299	0.476	-34.000	53.724	
151				-0.718	0.516	-0.679	0.488	-114.000	79.364	
152				-0.396	0.505	-0.375	0.477	-103.000	129.900	
153				0.027	0.295	0.026	0.290	0.600	6.620	
154				-0.139	0.295	-0.137	0.290	-8.000	16.912	
155				-0.011	0.295	-0.011	0.290	-0.100	2.759	
156				0.022	0.295	0.022	0.290	0.200	2.671	

	Study name	Comparison
157	Bondonno et al. 2020	Serial 7 subtractions correct
158	Bondonno et al. 2020	Serial 7 subtractions total
159	Evans et al. 2017	TMT B number of errors
160	Evans et al. 2017	TMT B time (s)
161	Evans et al. 2017	Visuospatial Working Memory double span
162	You et al. 2021	N1 back correct responses
163	You et al. 2021	N1 back correct responses time (s)
164	You et al. 2021	Stroop congruent correct response %
165	You et al. 2021	Stroop congruent correct response time
166	You et al. 2021	Stroop control correct response %
167	You et al. 2021	Stroop control correct response time
168	You et al. 2021	Stroop incongruent correct response %
169	You et al. 2021	Stroop incongruent correct response time
170	Whyte et al. 2021	Go No Go Accuracy
171	Whyte et al. 2021	Go No Go Accuracy beverage vs time
172	Whyte et al. 2021	Go No Go reaction time
173	Whyte et al. 2021	The Modified Attenti on Network Task (MANT)
174	Krikorian et al. 2022	Controlled Oral Word Association Test Category form
175	Krikorian et al. 2022	Controlled Oral Word Association Test phonemic access
176	Igwea et al. 2020	CategoCategory fluency at 4 weeks
177	Igwea et al. 2020	CategoCategory fluency at 8 weeks
178	Igwea et al. 2020	Letter fluency at 4 weeks
179	Igwea et al. 2020	Letter fluency at 8 weeks
180	Igwea et al. 2020	Stroop the time difference between the congruent and incongruent tasks at 4 weeks
181	Igwea et al. 2020	Stroop the time difference between the congruent and incongruent tasks at 8 weeks
182	Yahya et al. 2017	Comprehensive trail making test 1 week 3
183	Yahya et al. 2017	Comprehensive trail making test 1 week 6
184	Yahya et al. 2017	Comprehensive trail making test 2 week 3
185	Yahya et al. 2017	Comprehensive trail making test 2 week 6
186	Yahya et al. 2017	Comprehensive trail making test 3 week 3
187	Yahya et al. 2017	Comprehensive trail making test 3 week 6
188	Yahya et al. 2017	Comprehensive trail making test 4 week 3
189	Yahya et al. 2017	Comprehensive trail making test 4 week 6
190	Yahya et al. 2017	Comprehensive trail making test 5 week 3
191	Yahya et al. 2017	Comprehensive trail making test 5 week 6
192	Keane et al. 2016	Digit vigilance % 1 hour
193	Keane et al. 2016	Digit vigilance % 2 hour
194	Keane et al. 2016	Digit vigilance % 3 hour
195	Keane et al. 2016	Digit vigilance % 5 hour

	Study design	age	Polyphenol dose mg	Dose	Polyphenol group	Duration in days	Duration	Data format	Difference in means	Standard Error
157	CO	Over 60	4.890	Below	Flavonoid	0.166	Acute	Independent groups (means, SD's)		
158	CO	Over 60	4.890	Below	Flavonoid	0.166	Acute	Independent groups (means, SD's)		
159	BTW-P	Over 60	150.000	Below	Resveratrol	98.000	Chronic	Independent groups (means, SD's)		
160	BTW-P	Over 60	150.000	Below	Resveratrol	98.000	Chronic	Independent groups (means, SD's)		
161	BTW-P	Over 60	150.000	Below	Resveratrol	98.000	Chronic	Independent groups (means, SD's)		
162	BTW-P	Over 60	1.800	Below	Flavonoid	84.000	Chronic	Independent groups (means, SD's)		
163	BTW-P	Over 60	1.800	Below	Flavonoid	84.000	Chronic	Independent groups (means, SD's)		
164	BTW-P	Over 60	1.800	Below	Flavonoid	84.000	Chronic	Independent groups (means, SD's)		
165	BTW-P	Over 60	1.800	Below	Flavonoid	84.000	Chronic	Independent groups (means, SD's)		
166	BTW-P	Over 60	1.800	Below	Flavonoid	84.000	Chronic	Independent groups (means, SD's)		
167	BTW-P	Over 60	1.800	Below	Flavonoid	84.000	Chronic	Independent groups (means, SD's)		
168	BTW-P	Over 60	1.800	Below	Flavonoid	84.000	Chronic	Independent groups (means, SD's)		
169	BTW-P	Over 60	1.800	Below	Flavonoid	84.000	Chronic	Independent groups (means, SD's)		
170	CO	Below 60	450.000	Below	Flavonoid	0.330	Acute	Independent groups (Sample size, p)		
171	CO	Below 60	450.000	Below	Flavonoid	0.330	Acute	Independent groups (Sample size, p)		
172	CO	Below 60	450.000	Below	Flavonoid	0.330	Acute	Independent groups (Sample size, p)		
173	CO	Below 60	450.000	Below	Flavonoid	0.330	Acute	Independent groups (Sample size, p)		
174	BTW-P	Below 60	Not	Not	Unclassifia	84.000	Chronic	Independent groups (Sample size, p)		
175	BTW-P	Below 60	Not	Not	Unclassifia	84.000	Chronic	Independent groups (Sample size, p)		
176	CO	Over 60	7.500	Below	Flavonoid	56.000	Chronic	Independent groups (means, SD's)		
177	CO	Over 60	7.500	Below	Flavonoid	56.000	Chronic	Independent groups (means, SD's)		
178	CO	Over 60	7.500	Below	Flavonoid	56.000	Chronic	Independent groups (means, SD's)		
179	CO	Over 60	7.500	Below	Flavonoid	56.000	Chronic	Independent groups (means, SD's)		
180	CO	Over 60	7.500	Below	Flavonoid	56.000	Chronic	Independent groups (means, SD's)		
181	CO	Over 60	7.500	Below	Flavonoid	56.000	Chronic	Independent groups (means, SD's)		
182	BTW-P	Below 60	2.500	Below	Flavonoid	42.000	Chronic	Independent groups (means, SD's)		
183	BTW-P	Below 60	2.500	Below	Flavonoid	42.000	Chronic	Independent groups (means, SD's)		
184	BTW-P	Below 60	2.500	Below	Flavonoid	42.000	Chronic	Independent groups (means, SD's)		
185	BTW-P	Below 60	2.500	Below	Flavonoid	42.000	Chronic	Independent groups (means, SD's)		
186	BTW-P	Below 60	2.500	Below	Flavonoid	42.000	Chronic	Independent groups (means, SD's)		
187	BTW-P	Below 60	2.500	Below	Flavonoid	42.000	Chronic	Independent groups (means, SD's)		
188	BTW-P	Below 60	2.500	Below	Flavonoid	42.000	Chronic	Independent groups (means, SD's)		
189	BTW-P	Below 60	2.500	Below	Flavonoid	42.000	Chronic	Independent groups (means, SD's)		
190	BTW-P	Below 60	2.500	Below	Flavonoid	42.000	Chronic	Independent groups (means, SD's)		
191	BTW-P	Below 60	2.500	Below	Flavonoid	42.000	Chronic	Independent groups (means, SD's)		
192	CO	Below 60	13.700	Below	Flavonoid	0.042	Acute	Independent groups (means, SD's)		
193	CO	Below 60	13.700	Below	Flavonoid	0.083	Acute	Independent groups (means, SD's)		
194	CO	Below 60	13.700	Below	Flavonoid	0.125	Acute	Independent groups (means, SD's)		
195	CO	Below 60	13.700	Below	Flavonoid	0.208	Acute	Independent groups (means, SD's)		

	Intervention N (Optional)	Control N (Optional)	Effect direction	Std diff in means	Std Err	Hedges's g	Std Err	Difference in means	Std Err	X
157				-0.013	0.295	-0.013	0.290	-0.100	2.271	
158				0.067	0.295	0.065	0.290	0.500	2.215	
159				1.000	0.239	0.990	0.236	0.100	0.023	
160				0.645	0.231	0.638	0.229	1.900	0.664	
161				0.091	0.225	0.090	0.223	0.100	0.248	
162				1.687	0.521	1.616	0.499	12.130	3.216	
163				1.232	0.488	1.180	0.467	155.600	56.476	
164				1.745	0.525	1.671	0.503	11.000	2.819	
165				-3.305	0.688	-3.165	0.659	-537.290	72.705	
166				0.617	0.458	0.591	0.438	3.000	2.174	
167				0.878	0.468	0.841	0.448	69.310	35.290	
168				-0.741	0.462	-0.710	0.443	-4.500	2.715	
169				3.365	0.695	3.223	0.666	360.970	47.972	
170				-0.133	0.239	-0.131	0.237			
171				-0.215	0.240	-0.213	0.237			
172				0.477	0.242	0.472	0.240			
173										
174				-0.228	0.386	-0.221	0.375			
175				1.266	0.422	1.228	0.409			
176				-0.658	0.388	-0.638	0.377	-4.000	2.299	
177				-0.307	0.380	-0.298	0.369	-2.000	2.464	
178				-0.153	0.379	-0.149	0.367	-1.000	2.464	
179				0.167	0.379	0.162	0.368	1.000	2.268	
180				-0.181	0.379	-0.176	0.368	-1.000	2.087	
181				-0.200	0.379	-0.194	0.368	-1.000	1.890	
182				0.150	0.339	0.146	0.331	1.000	2.256	
183				0.241	0.339	0.235	0.332	2.200	3.087	
184				0.971	0.358	0.949	0.349	8.800	3.064	
185				0.214	0.339	0.210	0.331	2.500	3.942	
186				-0.059	0.338	-0.058	0.331	-0.600	3.415	
187				0.376	0.341	0.368	0.333	3.400	3.054	
188				0.052	0.338	0.051	0.331	0.500	3.241	
189				-0.168	0.339	-0.164	0.331	-1.400	2.814	
190				0.255	0.340	0.249	0.332	2.300	3.055	
191				0.335	0.341	0.327	0.333	2.600	2.625	
192				-0.025	0.272	-0.025	0.268	-0.170	1.818	
193				-0.071	0.272	-0.070	0.268	-0.500	1.917	
194				0.033	0.272	0.033	0.268	0.300	2.441	
195				0.017	0.272	0.017	0.268	0.180	2.843	

	Study name	Comparison
196	Keane et al. 2016	Digit vigilance RT 1 hour
197	Keane et al. 2016	Digit vigilance RT 2 hour
198	Keane et al. 2016	Digit vigilance RT 3 hour
199	Keane et al. 2016	Digit vigilance RT 5 hour
200	Keane et al. 2016	RVIP % 1 hour
201	Keane et al. 2016	RVIP % 2 hour
202	Keane et al. 2016	RVIP % 3 hour
203	Keane et al. 2016	RVIP % 5 hour
204	Keane et al. 2016	RVIP RT 1 hour
205	Keane et al. 2016	RVIP RT 2 hour
206	Keane et al. 2016	RVIP RT 3 hour
207	Keane et al. 2016	RVIP RT 5 hour
208	Keane et al. 2016	Stroop % 1 hour
209	Keane et al. 2016	Stroop % 2 hour
210	Keane et al. 2016	Stroop % 3 hour
211	Keane et al. 2016	Stroop % 5 hour
212	Keane et al. 2016	Stroop RT 1 hour
213	Keane et al. 2016	Stroop RT 2 hour
214	Keane et al. 2016	Stroop RT 3 hour
215	Keane et al. 2016	Stroop RT 5 hour

	Study design	age	Polyphenol dose mg	Dose	Polyphenol group	Duration in days	Duration	Data format	Difference in means	Standard Error
196	CO	Below 60	13.700	Below	Flavonoid	0.042	Acute	Independent groups (means, SD's)		
197	CO	Below 60	13.700	Below	Flavonoid	0.083	Acute	Independent groups (means, SD's)		
198	CO	Below 60	13.700	Below	Flavonoid	0.125	Acute	Independent groups (means, SD's)		
199	CO	Below 60	13.700	Below	Flavonoid	0.208	Acute	Independent groups (means, SD's)		
200	CO	Below 60	13.700	Below	Flavonoid	0.042	Acute	Independent groups (means, SD's)		
201	CO	Below 60	13.700	Below	Flavonoid	0.083	Acute	Independent groups (means, SD's)		
202	CO	Below 60	13.700	Below	Flavonoid	0.125	Acute	Independent groups (means, SD's)		
203	CO	Below 60	13.700	Below	Flavonoid	0.208	Acute	Independent groups (means, SD's)		
204	CO	Below 60	13.700	Below	Flavonoid	0.042	Acute	Independent groups (means, SD's)		
205	CO	Below 60	13.700	Below	Flavonoid	0.083	Acute	Independent groups (means, SD's)		
206	CO	Below 60	13.700	Below	Flavonoid	0.125	Acute	Independent groups (means, SD's)		
207	CO	Below 60	13.700	Below	Flavonoid	0.208	Acute	Independent groups (means, SD's)		
208	CO	Below 60	13.700	Below	Flavonoid	0.042	Acute	Independent groups (means, SD's)		
209	CO	Below 60	13.700	Below	Flavonoid	0.083	Acute	Independent groups (means, SD's)		
210	CO	Below 60	13.700	Below	Flavonoid	0.125	Acute	Independent groups (means, SD's)		
211	CO	Below 60	13.700	Below	Flavonoid	0.208	Acute	Independent groups (means, SD's)		
212	CO	Below 60	13.700	Below	Flavonoid	0.042	Acute	Independent groups (means, SD's)		
213	CO	Below 60	13.700	Below	Flavonoid	0.083	Acute	Independent groups (means, SD's)		
214	CO	Below 60	13.700	Below	Flavonoid	0.125	Acute	Independent groups (means, SD's)		
215	CO	Below 60	13.700	Below	Flavonoid	0.208	Acute	Independent groups (means, SD's)		

	Intervention N (Optional)	Control N (Optional)	Effect direction	Std diff in means	Std Err	Hedges's g	Std Err	Difference in means	Std Err	X
196				-0.018	0.272	-0.018	0.268	-0.830	12.427	
197				0.039	0.272	0.039	0.268	1.750	12.123	
198				-0.335	0.274	-0.330	0.270	-17.510	14.215	
199				-0.381	0.275	-0.375	0.271	-26.090	18.661	
200				-0.098	0.272	-0.097	0.268	-2.270	6.301	
201				-0.004	0.272	-0.004	0.268	-0.100	7.037	
202				-0.072	0.272	-0.071	0.268	-1.770	6.692	
203				0.006	0.272	0.006	0.268	0.160	6.739	
204				0.047	0.272	0.046	0.268	2.960	17.077	
205				-0.338	0.274	-0.333	0.270	-38.580	31.078	
206				-0.300	0.274	-0.296	0.270	-22.440	20.335	
207				0.036	0.272	0.036	0.268	2.610	19.678	
208										
209										
210										
211										
212										
213										
214										
215										
